# Circadian Phase Advances in Response to Weekend Morning Light in Adolescents With Short Sleep and Late Bedtimes on School Nights

**DOI:** 10.3389/fnins.2020.00099

**Published:** 2020-02-12

**Authors:** Ieva Misiunaite, Charmane I. Eastman, Stephanie J. Crowley

**Affiliations:** Biological Rhythms Research Laboratory, Department of Psychiatry and Behavioral Sciences, Rush University Medical Center, Chicago, IL, United States

**Keywords:** sleep, circadian rhythms, delayed sleep onset, adolescence, bright light treatment

## Abstract

Many adolescents fall asleep too late to get enough sleep (8–10 h) on school nights. Morning bright light advances circadian rhythms and could help adolescents fall asleep earlier. Morning bright light treatment before school, however, is difficult to fit into their morning schedule; weekends are more feasible. We examined phase advances in response to morning light treatment delivered over one weekend. Thirty-seven adolescents (16 males; 14.7–18.0 years) who reported short school-night sleep (≤7 h) and late bedtimes (school-nights ≥23:00; weekend/non-school nights ≥24:00) slept as usual at home for ∼2 weeks (“baseline”) and then kept a fixed sleep schedule (baseline school-night bed and wake-up times ±30 min) for ∼1 week before living in the lab for one weekend. Sleep behavior was measured with wrist actigraphy and sleep diary. On Saturday morning, we woke each participant 1 h after his/her midpoint of baseline weekend/non-school night sleep and 1 h earlier on Sunday. They remained in dim room light (∼20 lux) or received 1.5 or 2.5 h of intermittent morning bright light (∼6000 lux) on both mornings. The dim light melatonin onset (DLMO), a phase marker of the circadian timing system, was measured on Friday and Sunday evenings to compute the weekend circadian phase shift. The dim room light and 1.5-h bright light groups advanced the same amount (0.6 ± 0.4 and 0.6 ± 0.5 h). The 2.5-h bright light group advanced 1.0 ± 0.4 h, which was significantly more than the other groups. These data suggest that it is possible to phase advance the circadian clock of adolescents who have late bedtimes and short school-night sleep in one weekend using light that begins shortly after their sleep midpoint.

## Introduction

Sleep regulatory mechanisms undergo developmental changes that shift alertness later into the evening and night for older post-pubertal adolescents. The circadian timing system is later (phase delayed) (Carskadon et al., 1997, 2004; Crowley et al., 2014) and homeostatic sleep pressure accumulates at a slower rate during waking in more mature adolescents compared to their pre-pubertal peers (Jenni et al., 2005; Taylor et al., 2005). Evening activities (e.g., homework, part-time work, and media use) displace sleep and also contribute to late bedtimes and late sleep onset for many adolescents (Carskadon, 1989-1990, 2011; Knutson and Lauderdale, 2009; Cain and Gradisar, 2010). Older adolescents report bedtimes ranging from 22:30 to 01:00 (Giannotti et al., 2002; Thorleifsdottir et al., 2002; Yang et al., 2005; National Sleep Foundation [NSF], 2006; Beijamini et al., 2008; Crowley et al., 2014), and those with a diagnosis of delayed sleep–wake phase disorder (DSWPD) report average bedtimes of midnight to almost 03:00 (Gradisar et al., 2011a; Saxvig et al., 2014). Unfortunately, delayed sleep onsets of older adolescence occur at the same time when school starts early (Carskadon et al., 1998; National Sleep Foundation [NSF], 2006; Crowley et al., 2014). The average school-day wake time for a high schooler in the United States is between 6 and 6:30 am (Wolfson and Carskadon, 1998; Crowley et al., 2007, 2014; Short et al., 2017), and many other countries report school-day wake times of 07:00 or earlier (Gradisar et al., 2011b; Short et al., 2017). This pattern of early school-day wake times and late sleep onset puts older adolescents at an increased risk for getting less than the recommended 8–10 h of sleep per night (Hirshkowitz et al., 2015; Paruthi et al., 2016) on school nights, and less than the 9–9.25 h per night that is required for cognitive function/attention (Short et al., 2018) and for emotion regulation (Fuligni et al., 2017).

Shifting circadian rhythms earlier (phase advance) could help adolescents who are struggling to get sufficient sleep on school nights due to late sleep onset. We have used morning bright light and a gradual advance of sleep/dark to advance circadian rhythms in young adults (Burgess et al., 2003; Eastman et al., 2005; Revell et al., 2006; Smith et al., 2009; Crowley and Eastman, 2015). Studies that have thoroughly tested a morning bright light treatment to phase advance the adolescent circadian system, however, are limited. Moreover, feasibility of daily morning bright light treatment limit the utility of this strategy in an adolescent population because it requires them to fit light treatment into their morning routine before school for several days, a challenging task for teens who already have difficulty waking up in the morning on school days. The purpose of the current study was to determine whether bright light on two mornings of a weekend can advance the circadian system of late-sleeping adolescents, and determine the duration of bright light that is most effective.

Phase response curves (PRCs) to bright light (Honma and Honma, 1988; Czeisler et al., 1989; Khalsa et al., 2003; Kripke et al., 2007; St Hilaire et al., 2012; Crowley and Eastman, 2017) provide the optimal time for bright light to produce phase advances relative to an individual’s circadian phase. Timing bright light relative to the phase of the individual can produce the largest phase advances in a few days. Our PRC to bright light constructed in older adolescents (Crowley and Eastman, 2017) shows that bright light beginning shortly after the midpoint of sleep produces the largest advances. In the current study, we examined phase advances when bright light was individually timed relative to an adolescent’s midpoint of weekend/non-school night sleep on both mornings of one weekend during the school year. We used the midpoint of weekend/non-school night sleep because our PRC to bright light is based on summer (unconstrained) sleep times, which are more like weekend/non-school nights during the school year. We compared phase shifts of the DLMO in response to room light (∼20 lux), 1.5 h of bright light (∼6000 lux), and 2.5 h of bright light (∼6000 lux) individually timed relative to the midpoint of weekend/non-school night sleep. We hypothesized that the DLMO will phase advance more with 1.5 and 2.5 h of bright light compared to room light.

## Materials and Methods

### Ethics Statement

The study was approved by the Rush University Medical Center’s Institutional Review Board, in compliance with the Declaration of Helsinki (except for registration in a database). A parent of the participant provided written consent for the child to participate in the study, and the adolescent co-signed the consent form to acknowledge assent. Participants were paid for their participation.

### Participants

Forty-one adolescents (19 male) who were attending high school and between 14 and 18 years were enrolled in this study. We enrolled adolescents who were more likely to benefit from a phase advancing treatment strategy; therefore, in order to qualify for the study, adolescents had to report insufficient sleep on school nights (<7 h on average) and late school-night bedtimes (23:00 or later, on average). They also had to report late weekend/non-school night bedtimes (midnight or later, on average) and compensate for their school-night sleep restriction by sleeping, on average, at least 1 h more on weekend/non-school days. These criteria were assessed with the Sleep Habits Survey (Wolfson and Carskadon, 1998) and pre-study sleep logs. Participants were otherwise healthy and without a history of a developmental disorder, psychotic disorder, bipolar disorder, neurological disorder, psychopathology, metabolic disorder, chronic medical condition, infectious illness, or sleep disorder (restless legs syndrome, periodic limb movement disorder, obstructive sleep apnea, or narcolepsy) as reported by the participant and a parent/legal guardian. Participants did not report suicidal ideation, though some (*n* = 6) reported elevated depressive symptoms [scores > 16 on the Center for Epidemiological Studies-Depression (CES-D) scale] (Radloff, 1977). Participants were medication-free, except for two female participants who were taking an oral contraceptive. Participants did not work night shifts or travel beyond two time zones in the month before starting the study. Circadian phase preference was measured with the Morningness Questionnaire of Smith et al. (1989), which ranges in score from 13 (eveningness) to 55 (morningness). Mid-sleep on free days (corrected for weekend oversleep; MSFsc) and social jetlag (difference between midpoint of sleep on free days and non-free days) was assessed using the Munich Chronotype Questionnaire (Roenneberg et al., 2004). Participants self-reported their race/ancestry by choosing one of the following: White/Caucasian, Asian/Asian-American, Native American/Alaskan Native, Native Hawaiian/Pacific Islander, Black/African-American, multiracial, or specified another race/ancestry. They also self-reported their ethnicity by choosing either Not Hispanic/Latino or Hispanic/Latino. Adolescent reports of race and ethnicity were confirmed by a parent using the same response categories. Parents completed questionnaires asking about their child’s sleep behavior, medical history, and demographic information. Participants were not color blind or deficient as measured by the Ishihara Color Blindness test.

### Experimental Protocol

**FIGURE 1:**
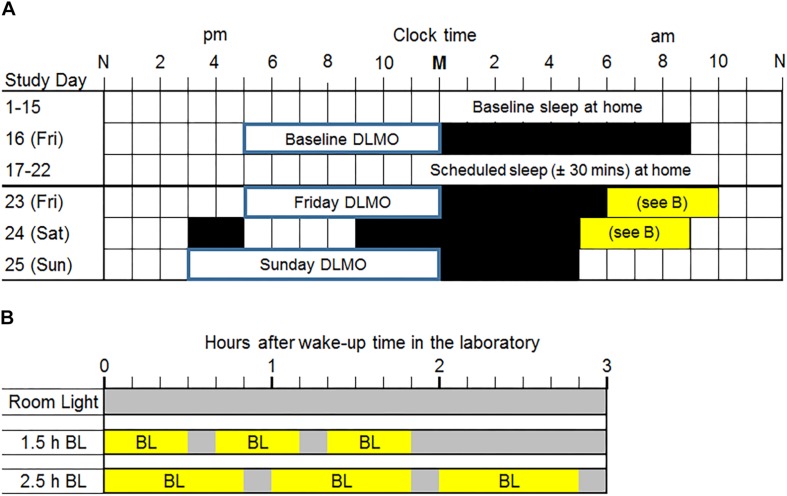
**(A)** Study protocol. M, midnight; N, noon. Two weeks of usual sleep at home (baseline days 1–15) was followed by a baseline dim light melatonin onset (DLMO) phase assessment in the laboratory on a Friday night (day 16). Participants were given a 9-h sleep opportunity in the laboratory after the baseline phase assessment. On days 17–22, all participants were prescribed a sleep schedule (±30 min) based on their average baseline school-night sleep onset and wake-up times measured from wrist actigraphy. Participants lived in the laboratory during the last weekend (days 23–25). DLMOs were measured on Friday (day 23) and Sunday (day 25) to compute weekend phase shifts. Black rectangles are scheduled sleep in the laboratory for a typical individual. Participants were awakened 1 h after their average baseline (days 1–15) weekend/non-school night mid-sleep time (rounded to the nearest half hour) on Saturday morning and an hour earlier on Sunday morning. This protocol shows an example of a weekend/non-school night mid-sleep time of 05:00 and thus a Saturday wake time of 6:00. Mid-sleep times ranged from 04:15 to 07:45; thus, Saturday wake times ranged from 05:15 to 08:45. On Saturday, all participants were given a 2-h nap opportunity in the afternoon to compensate for shortened sleep on Friday night. **(B)** Morning light exposure for each group. Within 5 min of waking on Saturday and Sunday mornings, participants had one of three morning light exposures. The room light group (top) received normal indoor lighting (gray bars; ∼20 lux) on both mornings. The 1.5 h bright light (BL; ∼6000 lux) group received three 30-min bright light exposures with 10 min of room light in between. The 2.5 h BL group received three 50-min bright light exposures with 10 min of room light in between.

Adolescents completed the 25-day protocol illustrated in Figure 1A during the school year (September–May) in Chicago, IL, United States (41°52′ N, 87°37′ W). For the first 15 days (baseline), participants slept at home on their usual sleep schedule. On Friday evening (Day 16), participants completed a circadian phase assessment in the laboratory to determine their Baseline DLMO, the most reliable marker of the central circadian clock (Klerman et al., 2002; Benloucif et al., 2005). The baseline phase assessment ended at their average baseline weekend/non-school night bedtime (to the nearest hour or half-hour clock time), and then they were put to bed. On Saturday morning, participants were awakened 9 h later and went home. Participants slept at home on days 17–22 with instructions to go to bed and wake up within ±30 min of their average baseline school-night bed and wake-up times. We kept adolescents on their baseline sleep schedule (±30 min) during this week to mimic what they would be doing at home before a weekend of bright light treatment would begin. The consistent sleep schedule should also stabilize the circadian system before timed bright light treatment occurred. On Friday (day 23), participants came to the lab after school and stayed all weekend for the weekend phase advancing protocol.

The weekend phase advancing protocol was individually timed and based on baseline sleep times derived from wrist actigraphy (described below). At the beginning of the weekend, participants completed another circadian phase assessment to determine their Friday DLMO (day 23) before the phase advancing protocol began. Saliva samples were collected beginning at 17:00 until each individual’s average weekend/non-school night bedtime (to the nearest hour or half-hour clock time), shortly after which they went to sleep in one of the private laboratory bedrooms. The next morning (Saturday), participants were awakened 1 h (±15 min) after their average weekend/non-school night mid-sleep time to either sit in normal room light or begin the morning light exposure as this time produces phase advances according to the adolescent PRC to bright light (Crowley and Eastman, 2017). On Sunday morning, we woke participants 1 h earlier than on Saturday morning. On Saturday (day 24), all participants were given a 2-h nap opportunity in the afternoon to compensate for shortened sleep on Friday night. Figure 1A shows an example of an adolescent with a weekend/non-school night mid-sleep time of 05:00; therefore, Saturday morning wake-up time was 06:00 and Sunday morning wake-up time was 05:00. Scheduled times varied among participants; wake-up times on Saturday morning ranged from 05:15 to 08:45. On Saturday night, participants were put to bed 8 h before their scheduled wake-up time on Sunday morning. At the end of the weekend (day 25), participants completed a circadian phase assessment from 15:00 to their own average weekend/non-school night bedtime (to the nearest hour or half-hour clock time) to determine Sunday DLMO, after which the participants slept in the laboratory.

**FIGURE 2:**
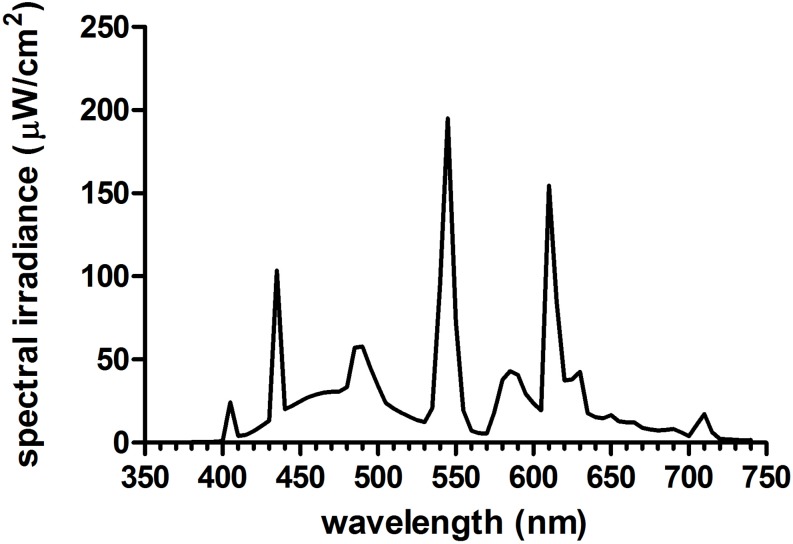
Spectral power distribution of the bright light boxes (Philips EnergyLight^®^) used in the current study. See Supplementary Material for source irradiance data and Table 1 for a-opic effective illuminances (a-opic lux) and a-opic equivalent daylight (D65) illuminances (a-opic EDI).

Figure 1B illustrates the time of morning light relative to wake-up time in the laboratory for each group on both weekend mornings. The room light group received normal indoor lighting (21 ± 6 lux) from ceiling light fixtures dimmed to the lowest setting from a control room. The 1.5 h bright light group received three 30-min bright light (5961 ± 254 lux) exposures with two 10-min breaks in room light in between. The 2.5 h bright light group received three 50-min bright light (5910 ± 142 lux) exposures with two 10-min breaks in room light in between. In the bright light groups, two light boxes (EnergyLight^®^, Philips Consumer Lifestyle, Drachten, Netherlands; 5000 K; screen size of 33 × 47 cm) were turned on within 5 min of waking. Figure 2 illustrates the spectral power distribution of the EnergyLights (see the Supplementary Material for source data). Retinal photoreceptor-weighted ɑ-opic effective illuminances (ɑ-opic lux) as described by Lucas et al. (2014) and ɑ-opic equivalent daylight (D65) illuminances (ɑ-opic EDI) computed according to international standard (CIE 026/E:2018) are reported in Table 1. Participants did not have access to their cell phones during the morning light sessions; however, they were allowed to watch movies or TV shows, access social media, or complete homework on a computer. The two light boxes were positioned on either side of the computer screen. The middle of the light box screens were 36 ± 4 cm from participants’ eyes, on average. Study staff measured illuminance levels at the level of both eyes with a light meter (ExTech EasyView 33^TM^, Extech Instruments, Waltham, MA, United States) every 15–25 min during morning bright light and room light exposures. Participants remained in normal indoor lighting for the remainder of the day.

**TABLE 1:** Human retinal photoreceptor weighted a-opic effective illuminances (a-opic lux) and a-opic equivalent daylight (D65) illuminances (a-opic EDI) for this study’s bright light boxes based on a 32-year-old standard observer.

**Photoreceptor**	**Photopigment**	**a-opic lux^1^**	**a-opic EDI^2^**
Short-wavelength (S) cones	S-cone photopsin (cyanolabe)	4238	4094
Medium-wavelength (M) cones	M-cone photopsin (chlorolabe)	5712	5546
Long-wavelength (L) cones	L-cone photopsin (erythrolabe)	5800	5918
Intrinsically photosensitive retinal ganglion cells (ipRGCs)	Melanopsin	5114	4634
Rods	Rhodopsin	5432	4901

*^1^Computed using the irradiance toolbox of Lucas et al. (2014). ^2^International standard CIE 026/E:2018; a-opic equivalent daylight illuminance (EDI) values (in lux) provide the illuminance level of daylight D65 (phase of daylight with a correlated color temperature of ∼6500 K) that produces an identical a-opic irradiance as the light box.*

Participants ran in cohorts of two or three and were assigned to the same morning light group. Groups were not randomly scheduled; the majority of the bright light groups were run first. Participants were assigned as they were eligible and available.

### Dim Light Melatonin Onset Phase Assessments

During the circadian phase assessments, salivary melatonin concentration was measured from approximately 2 ml of saliva collected every 30 min using Salivettes (Sarstedt, Nümbrecht, Germany). Participants remained awake in dim light (<5 lux) sitting in comfortable recliners, except when they needed to use the attached washroom (also <5 lux). They were not allowed to eat or drink in the 10 min before each sample and washroom trips were not allowed during this time. Saliva samples were immediately centrifuged after collection and frozen. These samples were later radioimmunoassayed (RIA) for melatonin concentration using commercially available kits (ALPCO, Salem, NH, United States) by SolidPhase, Inc. (Portland, ME, United States). An individual’s samples were analyzed in the same batch. The intra-assay coefficients of variation for low (evening) and high (nighttime) levels of salivary melatonin were 4.1 and 4.8%, respectively. The inter-assay coefficients of variation for low and high levels of salivary melatonin were 6.6 and 8.4%, respectively. DLMO phase, expressed in a 24-h clock time, was determined by linear interpolation across the time points before and after the melatonin concentration increased to and stayed above 4 pg/ml (Carskadon et al., 1997; Crowley et al., 2016).

### Actigraphic Sleep

Participants wore an actigraph (Actiwatch Spectrum, Philips Respironics, Inc., Bend, OR, United States) on their non-dominant wrist and completed daily sleep logs to record bedtime, sleep onset time, wake time, and whether they attended school or not. Participants also telephoned daily to a time-stamped voicemail messaging system at bedtime and wake time. Participants visited the laboratory every 2–3 days so that we could download the actigraphy data and review sleep logs with them; participants were questioned about any inconsistencies between the actogram and sleep logs.

Wrist activity data were analyzed using the Actiware 6 software (version 6.0.9, Philips Respironics, Inc., Bend, OR, United States) to estimate sleep/wake using the low wake threshold and the sleep epochs sleep interval detection algorithm. We chose the low threshold based on the work of Meltzer et al. (2012), who showed that the low threshold has the highest sensitivity and specificity to detect sleep and wake in 13–18 year olds. Each sleep episode was manually inspected within a rest interval beginning 15 min before the participants’ reported bedtime and ending 15 min after their reported wake-up time on their daily sleep log. The first of three consecutive 1-min epochs of sleep defined sleep onset and the first epoch after the last five consecutive 1-min epochs of sleep defined wake-up time. Total sleep time was defined as the amount of time scored as sleep between sleep onset time and wake-up time. Mid-sleep time was defined as the midpoint between sleep onset and wake time.

### Statistical Analyses

The distribution of weekend phase shifts was first inspected to verify assumptions of normality (skew = 0.10; kurtosis = −0.63) and heterogeneity of variance among groups [*F*(2,34 = 0.04, *p* = 0.96]. Then, a one-way analysis of variance with weekend phase shifts (Friday DLMO to Sunday DLMO) as the outcome and group as the between-subjects factor tested our primary hypothesis. Group differences were determined using Tukey’s adjustment for multiple comparisons. Effect sizes (Cohen’s *d*) were also computed and evaluated with the standard descriptors (small effect = 0.2, medium effect = 0.5, and large effect = 0.8). Analyses were run with baseline DLMO, Friday DLMO, and depression symptoms measured by the CES-D as covariates, but this did not change the results, so they are not reported here. Phase shift responses were variable; therefore, we also computed the proportion of participants who shifted by >1 h in each group and compared these proportions using a chi-square test.

Actigraphic sleep/wake data collected during baseline (days 1–15) were examined to verify that the sleep inclusion criteria were met. Sleep onset time, wake-up time, and total sleep time measured from wrist actigraphy were averaged separately for school nights and for weekend/non-school nights for each participant. School and weekend/non-school nights were defined by whether the adolescent reported attending school the next day or not on their daily sleep diary. A 3 (group: room light, 1.5 h bright light, 2.5 h bright light)-by-2 (night type: school night vs. weekend/non-school night) analysis of variance tested for sleep timing and duration differences among the groups, by the type of day that followed, and whether differences between school and weekend/non-school nights differed by group assignment. Follow-up one-way ANOVAs with Tukey’s *post hoc* tests to account for multiple comparisons examined group differences in school nights and weekend/non-school nights separately.

Finally, we examined sleep onset, wake-up time, and total sleep time on scheduled days 17–22 to exclude the possibility that sleep in the week proximal to the weekend phase advancing protocol contributed to the phase shift results. School nights and weekend/non-school nights were not separated because participants were instructed to keep the same sleep schedule regardless of whether they attended school the next day or not. A one-way analysis of variance and Tukey’s *post hoc* tests examined whether sleep differed among the groups in the week before the weekend spent in the laboratory.

## Results

### Participants

Three males and one female decided to discontinue the study; therefore, 37 adolescents (16 males) completed the study. Demographic information for the participants that completed the study is in Table 2. Groups did not differ by age [*F*(2,36) = 0.14, *p* = 0.87], morningness score [*F*(2,36) = 0.63, *p* = 0.54], MSFsc [*F*(2,34) = 0.14, *p* = 0.87], or social jetlag [*F*(2,36) = 1.15, *p* = 0.33]. The distribution of participants in each group did not differ by sex [χ^2^(2) = 0.30, *p* = 0.86], ancestry [African-American versus other ancestries: χ^2^(2) = 1.4, *p* = 0.50], or ethnicity [χ^2^(2) = 4.1, *p* = 0.13]. Those who decided to discontinue the study (two non-Hispanic White, one Hispanic White, and one Hispanic multi-racial participants) were similar in age [15.7 ± 0.91 years; *t*(39) = 1.31, *p* = 0.20], morningness score [37.25 ± 5.5; *t*(39) = −1.42, *p* = 0.16], MSFsc [5:20 ± 1:44; *t*(37) = 0.28, *p* = 0.78], and social jetlag [2.7 ± 1.2 h; *t*(39) = 0.88, *p* = 0.38] as participants that completed the study. Three participants discontinued the study during baseline and one participant assigned to the room light group decided to discontinue the study during the laboratory weekend.

**TABLE 2:** Participant demographics.

	**Room light**	**1.5 h bright light**	**2.5 h bright light**
*N*	13	11	13
Age in years (mean ± SD)	16.4 ± 1.1	16.5 ± 0.9	16.3 ± 1.2
(Minimum−maximum)	(14.7−18)	(15.3−18)	(14.9−18)
**Sex (*N*)**			
Females	7	7	7
Males	6	4	6
**Ancestry (*N*)**			
African-American	3	2	5
White	7	3	5
Asian	1	0	0
Multiple	0	3	2
Other	2	3	1
**Ethnicity (*N*)**			
Non-Hispanic	11	5	8
Hispanic	2	6	5
Morningness score^a^ (mean ± SD)	32.2 ± 4.9	34.3 ± 5.6	34.0 ± 5.0
Mid-sleep time on free days^b^ (mean ± SD)	5:36 ± 0:52	5:30 ± 1:05	5:24 ± 0:54
Social jetlag (hours)^b^ (mean ± SD)	3.3 ± 0.8	2.8 ± 0.7	3.1 ± 0.7

*^*a*^Smith Morningness Questionnaire. ^*b*^Computed from the Munich Chronotype Questionnaire (MCTQ) and corrected for weekend oversleep. Two participants reported waking with an alarm clock on free/weekend days and therefore were not included in the means and SDs reported for the room light (*n* = 12) and the 2.5 h bright light (*n* = 12) groups.*

### Circadian Phase and Phase Shifts

**FIGURE 3:**
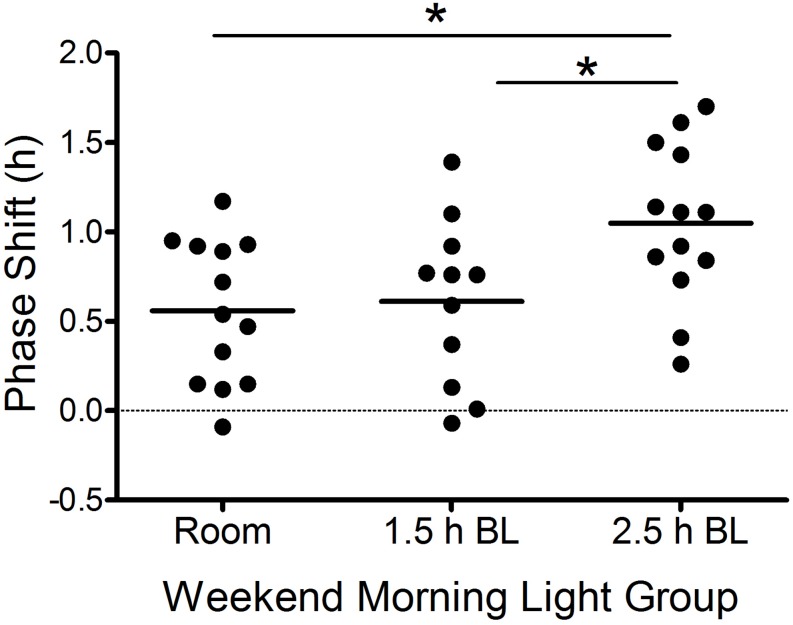
Weekend phase shifts [Friday DLMO (day 23) to Sunday DLMO (day 25)] in response to the weekend morning light conditions in each group. Each point represents an individual participant. Horizontal lines are group means. Positive numbers represent phase advances. BL, bright light. *p ≤ 0.05.

Table 3 shows average Baseline (day 16), Friday (day 23), and Sunday (day 25) DLMOs for each group. There were no group differences in Baseline DLMOs [*F*(2,32) = 1.2, *p* = 0.30] or Friday DLMOs before the phase shifting protocol [*F*(2,34) = 0.7, *p* = 0.49]. Individual and mean weekend phase shifts (Friday DLMO – Sunday DLMO) in response to the three weekend morning light conditions are shown in Figure 3 and Table 3. Weekend phase shifts were significantly different among the three groups [*F*(2,34) = 4.9, *p* = 0.02]. The room light group and 1.5 h bright light group both advanced by 0.6 h; phase shifts did not differ between these two groups (*p* = 0.95; *d* = 0.12). The 2.5 h bright light group, however, shifted by 1.0 h, on average, and this shift was significantly greater than both the room light (*p* = 0.02; *d* = 1.16) and the 1.5 h bright light (*p* = 0.05; *d* = 0.96) groups. A greater proportion of the participants in the 2.5 h BL group [7 of 13 (54%)] shifted in the advance direction by 1 h or more compared to the other groups [room light: 1 of 13 (8%); 1.5 h BL: 2 of 11 (18%)], χ^2^(2) = 7.6, *p* = 0.02.

**TABLE 3:** Circadian phase, phase shifts, and timing of morning light (mean ± SD).

	**Room light**	**1.5 h Bright light**	**2.5 h Bright light**
*N*	13	11	13
Baseline DLMO (day 16)^a^	21:35 ± 1:08	21:19 ± 1:20	22:11 ± 1:29
Friday DLMO (day 23)	21:17 ± 1:06	21:14 ± 1:28	21:47 ± 1:14
Sunday DLMO (day 25)	20:44 ± 1:17	20:38 ± 1:37	20:44 ± 1:19
Friday (day 23)–Sunday (day 25) phase advance (h)	0.6 ± 0.4	0.6 ± 0.5	1.0 ± 0.4*
Interval from Friday (day 23) DLMO to Saturday morning wake/start of bright light (h)	9.0 ± 1.0	9.0 ± 1.4	9.1 ± 1.3
Time of first morning light (Saturday)^b^	6:17 ± 0:58	6:15 ± 0:40	6:53 ± 0:53

* Different from room light (*p* = 0.02) and 1.5 h bright light (*p* = 0.05) groups. ^*a*^*N* = 10 in 1.5 h bright light group and *N* = 12 in the 2.5 h bright light group because melatonin levels did not exceed 4 pg/ml before the end of sampling during the baseline phase assessment (day 16). ^*b*^Scheduled wake-up time in the laboratory on Saturday morning, which was ∼1 h after baseline (days 1–15) weekend/non-school night mid-sleep times; Sunday wake time was scheduled 1 h earlier. Bright light exposure in the 1.5 and 2.5 h bright light groups started within 5 min of wake time on both mornings.

To confirm that the three groups were exposed to the morning light at the same circadian time, we computed the time interval from Friday DLMO to the scheduled wake-up time (the time when lights were turned on) on Saturday (Table 3). The time that participants woke up and were exposed to light relative to their Friday DLMO was similar across groups [*F*(2,34) = 0.03, *p* = 0.98]. Bright light started about 9 h after Friday DLMO, on average, which corresponds to the approximate peak of the phase advancing portion of the adolescent PRC to bright light (Crowley and Eastman, 2017). Supplementary Figure S1 shows when room or bright light exposure began for each participant and their corresponding weekend phase shift response.

### Behavioral Sleep

Participants were eligible for the study if they reported late bedtimes and short school-night sleep duration. Actigraphy data from baseline days 1–15 reflect these inclusion criteria for sleep (Table 4). Average sleep onset times were after midnight on school nights in all three groups. The 2.5 h bright light group averaged the latest sleep onset time on both school nights and weekend/non-school nights, but this was not statistically different from the other groups. Baseline school-night wake-up times were at about 6:30 am and did not differ among groups. Average school-night total sleep times measured from actigraphy at the low threshold were 4.8–5.5 h, on average, with the 2.5-h bright light group sleeping the least. On weekend/non-school nights during baseline (days 1–15), average sleep onset was about an hour later, wake-up time was about 2.5 h later, and total sleep time was over an hour longer compared to school nights. These differences between school nights and weekend/non-school nights were consistent among the groups.

**TABLE 4:** Sleep times from wrist actigraphy (mean ± SD).

	**Room light**	**1.5 h Bright light**	**2.5 h Bright light**	**Group ANOVA**
*N*	13	11	13	

**Baseline sleep (days 1–15)^a,b^**				
**School nights**				
Sleep onset time	00:03 ± 0:37	00:08 ± 0:54	00:48 ± 1:04	*F*(2,34) = 2.81, *p* = 0.07
Wake-up time	06:32 ± 0:29	06:32 ± 0:27	06:25 ± 0:39	*F*(2,34) = 0.22, *p* = 0.80
Total sleep time (h)	5.5 ± 0.6	5.5 ± 0.7	4.8 ± 0.8*	*F*(2,34) = 4.50, *p* = 0.02
**Weekend/non-school nights**				
Sleep onset time	01:13 ± 0:34	01:02 ± 0:48	01:47 ± 1:07	*F*(2,34) = 2.56, *p* = 0.09
Wake-up time	09:08 ± 1:16	09:20 ± 1:15	09:33 ± 1:07	*F*(2,34) = 0.40, *p* = 0.67
Total sleep time (h)	6.7 ± 1.0	6.7 ± 1.1	6.6 ± 1.0	*F*(2,34) = 0.10, *p* = 0.92

**Scheduled sleep (days 17–22)^c^**				
Sleep onset time	00:12 ± 0:35	00:10 ± 0:50	00:42 ± 0:51	*F*(2,34) = 1.88, *p* = 0.17
Wake-up time	06:52 ± 0:35	07:13 ± 0:45	06:56 ± 0:47	*F*(2,34) = 0.82, *p* = 0.45
Total sleep time (h)	5.7 ± 0.5	5.8 ± 0.7	5.3 ± 0.4	*F*(2,34) = 3.02, *p* = 0.06

**Laboratory weekend sleep (days 23–24)**				
**Friday to Saturday**				
Sleep onset time	01:17 ± 0:53	01:07 ± 0:46	01:50 ± 0:51	*F*(2,34) = 2.47, *p* = 0.10
Wake-up time	06:13 ± 0:57	06:11 ± 0:40	06:49 ± 0:52	*F*(2,34) = 2.18, *p* = 0.13
Total sleep time (h)	4.5 ± 0.4	4.4 ± 0.5	4.6 ± 0.3	*F*(2,34) = 1.10, *p* = 0.34
**Saturday (day 24) nap**				
Sleep onset time	15:24 ± 1:06	15:18 ± 0:40	15:55 ± 0:55	*F*(2,34) = 1.60, *p* = 0.22
Wake-up time	17:15 ± 0:56	17:14 ± 0:41	17:49 ± 0:54	*F*(2,34) = 1.95, *p* = 0.16
Total sleep time (h)	1.7 ± 0.3	1.7 ± 0.1	1.8 ± 0.1	*F*(2,34) = 0.50, *p* = 0.61
**Saturday to Sunday**				
Sleep onset time	21:55 ± 1:14	21:31 ± 0:46	22:09 ± 0:58	*F*(2,34) = 1.15, *p* = 0.33
Wake-up time	05:13 ± 0:59	05:11 ± 0:41	05:35 ± 0:54	*F*(2,34) = 1.97, *p* = 0.16
Total sleep time (h)	6.5 ± 0.7	6.4 ± 0.4	6.6 ± 0.4	*F*(2,34) = 0.40, *p* = 0.68

^*a*^During baseline, participants were asked to sleep as they usually do on school nights and weekend/non-school nights. ^*b*^Sleep onset time [*F*(1,68) = 25.32, *p* < 0.01] and wake-up time [*F*(1,68) = 168.60, *p* < 0.01] were later on non-school nights compared to school nights during baseline days. This delay in sleep timing did not differ among groups. Total sleep time was longer on weekends/non-school nights compared to school nights [*F*(1,68) = 168.60, *p* < 0.01] for all three groups. ^*c*^On days 17–22 (school nights and weekend/non-school nights), participants were asked to sleep at scheduled times (±30 min) that were prescribed based on their average self-selected school-night sleep times from baseline days 1–12. Sleep timing and duration did not differ among groups. * 2.5 h Bright light < room light (*p* = 0.02); trend for 2.5 h bright light < 1.5 h bright light (*p* = 0.07).

Scheduled sleep on days 17–22 was designed to be similar to school-night sleep during baseline (±30 min), and actigraphic data in Table 4 show that participants did well, on average, following these instructions. Sleep timing and duration did not differ among the three groups during this week before to the phase advancing protocol in the laboratory.

## Discussion

This is the first study to test different durations of bright light designed to phase advance the circadian system of adolescents over one weekend. Additionally, this study is the first to test a weekend phase advancing protocol with an objective measure of circadian phase (i.e., DLMO) to examine how effective the bright light stimuli were in producing phase advances in just 2 days. Unexpectedly, our data indicate that the 1.5 h morning bright light exposure pattern (three 30-min exposures of ∼6000 lux spread over almost 2 h) on Saturday and Sunday mornings is not any more effective than remaining in room light to phase advance adolescents circadian rhythms. Of note, transitioning from sleep/dark to normal room light on both weekend mornings produced a phase advance of >30 min. The 2.5 h of morning bright light spread out over almost 3 h on the two weekend mornings phase advanced the DLMO by 1 h, which was significantly greater than the other two groups. The early bedtime on Saturday night in the dark laboratory bedroom presumably contributed to the phase advances in all three groups.

Two previous studies have examined weekend bright light exposure to advance the circadian rhythms of adolescents (Crowley and Carskadon, 2010; Bonnar et al., 2015). One of these studies combined weekend bright light exposure with a 4-week sleep education program to improve sleep knowledge and increase motivation to change problematic sleep behaviors (e.g., weekend sleep-ins) with or without parental involvement in the sleep education process (Bonnar et al., 2015). The intervention groups tested were parental involvement alone, weekend morning bright light alone, or both and were compared to a class-as-usual control. On the third weekend, adolescents who received bright light were instructed to wake up 30 min earlier than usual on Saturday and another 30 min earlier on Sunday morning. Upon waking on these mornings, they were instructed to wear a portable light device (“Re-timer”) that emits short wavelength light (500 nm, 506 lux) from LEDs attached to a pair of glasses for 30–60 min. Adolescents reported wearing the Re-timers for an average duration of 29 min on Saturday mornings and 34 min on Sunday mornings. Overall, all intervention groups experienced a ∼15-min reduction in self-reported sleep onset latency and a ∼27-min increase in self-reported total sleep time on school nights, but not an earlier school-night bedtime compared to the control group. These self-reported sleep improvements were even greater for a subclinical group identified as having delayed sleep timing. Self-reported sleep times of adolescents who received light on two weekend mornings, however, did not differ from the intervention group without the light visor, suggesting that ∼30 min of short wavelength light timed 30–60 min before usual weekend wake-up time did not provide additional benefits in advancing sleep timing. Our current study’s results would suggest that 30 min of morning light was too short, but it could also be that the light was timed too late or the delivery system of the light was not effective. Compliance with light treatment on weekend mornings may have also contributed to the lack of differences seen between the groups; 74% of participants reported using the Re-timers on Saturday and 67% on Sunday. Finally, whether or not the light produced a circadian phase advance over a weekend is unknown because there was no objective measure of circadian phase (e.g., DLMO).

The second study that used bright light exposure on weekend mornings tested whether adding bright light to a typical late weekend sleep schedule could prevent circadian rhythms from shifting later (delaying) over the weekend in adolescents aged 15–16 years (Crowley and Carskadon, 2010). All participants were instructed to go to bed 1.5 h later and wake 3 h later on a weekend compared to their school-night schedule to mimic the average weekend sleep pattern of an adolescent. The morning light group was instructed to sit in front of a small LED-based short wavelength light box for 1 h on both weekend mornings shortly after waking and another group was not given a light box. Results showed that circadian phase measured from the DLMO on Friday evening and again on Sunday evening delayed by 46 ± 36 min after keeping a late weekend sleep schedule and adding morning short wavelength light after the late wake time did not prevent the delay shift from occurring; the morning light group also delayed by 38 ± 28 min and did not differ from the group without light. The lack of differences between the groups may be due to the small light box not covering the entire visual field (the screen measured 11 × 6.5 cm), the light duration was too short, or that the light was timed too late. *Post hoc* analyses showed that the morning short wavelength light exposure started 11.5–14 h after baseline DLMO, which was too late to target the phase advance region of the adolescent PRC to white light (Crowley and Eastman, 2017). Weekend wake times and bright light exposure needed to occur earlier for the light to phase advance or stabilize the circadian clock in a short period of time. In the current study, we used the midpoint of weekend/non-school night sleep to time the bright light, which successfully targeted the phase advance region of the adolescent PRC to bright light (Crowley and Eastman, 2017). Light started about 7–11 h after Friday DLMO (Supplementary Figure S1). Although this method requires very early wake times on the weekend, our method of using the midpoint of sleep is more efficient as it requires fewer days (one weekend) to shift the system earlier.

The amount of inter-individual variability in responses to light is notable, but similar to the adolescent PRC to light (Crowley and Eastman, 2017). For example, when the 2.5-h bright light exposure started approximately 9 h after Friday DLMO, responses varied by more than an hour (Supplementary Figure S1). Puberty stage may contribute to this variability in light sensitivity as seen previously (Crowley et al., 2015), though the restricted age range of the current sample of adolescents makes this less likely. Other individual factors, such as light exposure history before the laboratory weekend (Hebert et al., 2002; Chang et al., 2011) or functional differences at various levels of light input to the SCN may be more likely to contribute to these differences.

Past studies have successfully used morning bright light to advance circadian rhythms in adults (Rimmer et al., 2000; Burgess et al., 2003; Eastman et al., 2005; Revell et al., 2006; Paul et al., 2009; Smith et al., 2009; Burke et al., 2013; Crowley and Eastman, 2015), though the duration of light exposure, the number of days of light exposure, and the pattern of light exposure (continuous vs. intermittent) differed among these studies. Furthermore, some studies tested the combination of afternoon melatonin and morning bright light. Of relevance to the current study, our laboratory tested different durations of morning bright light given over 3 days of a gradually advancing sleep/dark schedule and 0.5 mg of afternoon melatonin to phase advance circadian rhythms in adults aged 18–40 years (Crowley and Eastman, 2015). All groups received afternoon melatonin and a 1 h/day advance of sleep/dark, and only differed by the morning light pattern: four 30-min exposures spread over 3.5 h (“2 h group”), four 15-min exposures spread over 3.25 h (“1-h group”), and one 30-min exposure (“0.5 h group”). The 2 h bright light group showed the largest phase advance (2.4 ± 0.8 h), but the 1 and 0.5 h bright light patterns also produced large phase shifts (1.7 ± 0.7 and 1.8 ± 0.8 h, respectively). It should be noted that the Crowley and Eastman study had three treatment days instead of two and also included afternoon melatonin, which likely contributed to the larger phase shifts than the current study.

Interestingly in the present study, 2.5 h of bright light produced a larger phase advance than room light, but 1.5 h of bright light did not advance adolescents any more than room light. The two bright light conditions differed not only in the total minutes of bright light exposure (1.5 and 2.5 h), but in the interval of time covered (∼2 vs. 3 h, Figure 1B). A longer interval can cover more of the advance region of the PRC to bright light. It is possible that extending the current study’s 1.5 h light pattern to occur over ∼3 h like the 2.5 h group would produce larger phase shifts than what we observed when it was spread over ∼2 h. For example, three 30-min exposures (1.5 h total) with 45 min of room light in between would cover 3 h and may even be more feasible for incorporating morning activities such as showering and preparing breakfast. This hypothesis would need to be systematically tested. Adolescents in the room light condition of the current study advanced by about 30 min in response to shifting their sleep/dark schedule earlier over just 2 days. This finding mimics our previous studies in adults (Burgess et al., 2003; Crowley and Eastman, 2013), and confirms that shifting sleep/dark also contributes to phase advancing the circadian system of adolescents.

When considering light treatment feasibility, it is important to note the limitations of traditional light boxes. Light boxes require the individual to maintain a relatively fixed position while sitting in front of the light boxes, especially when using one very small light box. This can often mean that activities are restricted during the treatment period. We used two of what are considered medium size light boxes. Additionally, the efficacy of light boxes for shifting circadian rhythms can depend on the size of the illuminated area, eye-screen distance, gaze direction, and head position (Dawson and Campbell, 1990; Eastman, 2011; Lau et al., 2018). Using alternative light sources, like head-mounted light sources or visors that emit short wavelength light to the eyes for a longer duration of time than has been previously tested (Bonnar et al., 2015) may increase the acceptability and feasibility of our weekend phase advancing protocol with morning light, especially for adolescents. Head-mounted light devices and visors, however, need to be tested to determine whether or not they work as well as the traditional light boxes, and what duration dose is effective and safe for young eyes to phase advance rhythms in one weekend. Finally, encouraging adolescents to go outdoors into sunlight first thing in the morning and engaging in a preferred weekend activity (e.g., sport, walking, etc.) could also increase feasibility of morning light exposure for some teens.

There are several ways to improve our weekend phase advancing protocol for adolescents who fall asleep late and get too little sleep on school nights. First, the weekend bright light treatment protocol may be more effective when combined with earlier bedtimes because light exposure in the hours just before habitual bedtime can produce phase delay shifts (Crowley and Eastman, 2017). We were unable to incorporate an earlier bedtime on both weekend nights in the current study because the phase assessment on Friday kept participants awake until their weekend/non-school night bedtime to complete salivary melatonin sampling. We did, however, set an earlier bedtime on Saturday night. Another strategy would be to test whether dimming evening light by wearing sunglasses for a few hours in the evening before bedtime can further advance rhythms. Second, the advance shifts produced by the weekend protocol can only be maintained if the adolescent keeps a consistent and early bedtime and wake time in the subsequent weeks. Behavioral strategies (e.g., motivational interviewing techniques) that facilitate earlier bedtimes during the school week following the bright light treatment could maintain gains and may even further advance their circadian rhythms. These additional strategies are currently being studied in our laboratory.

The aim of the current study was to test durations of bright light exposure on two mornings of a weekend to determine the most effective method to “jump start” an advance shift in circadian phase. Therefore, we limited our main outcome to the weekend DLMO phase shift and did not collect sleep data after the weekend phase advancing protocol to determine how sleep/wake behavior may have changed. We can only speculate that by shifting the central circadian clock earlier, we likely shifted the physiologic ability to fall asleep earlier too in these adolescent participants. Previous studies suggest that the magnitude of shift in phase and sleep onset may not be linear. Wright et al. (2013), for example, found that the DLMO of adults in their 20 and 30 s advanced by ∼2 h in natural light conditions of camping in Colorado compared to modern electric light exposure, but sleep onset advanced slightly less (∼1.2 h). If we assume a similar relationship in our study of adolescents, a 1-h advance in DLMO (mean of 2.5 h bright light group) would produce a ∼36-min advance in sleep onset. Saxvig et al. (2014) studied adolescents (16–25 years) diagnosed with DSWPD in four treatment groups: dim red light + placebo pill, bright light + placebo pill, dim red light + melatonin pill, and bright light + melatonin pill. Wake-up times were gradually advanced over 2 weeks until their preferred time. In all groups, DLMO advanced ∼2 h, self-reported bedtime advanced 1 h and wake-up time advanced by 2 h. Assuming a similar relationship between DLMO phase advance and bedtime, a 1 h advance in DLMO would produce a ∼30-min advance in bedtime. Based on these studies, our weekend phase advancing protocol with 2.5 h of morning bright light could advance sleep onset by at least 30–36 min on school nights, on average, and may increase school-night sleep duration by 2.5–3 h over a school week. We are currently testing the short and long-term changes in sleep behavior and cognitive function after the weekend phase advancing protocol with 2.5 h of bright light.

External factors that heighten alertness in the evening (e.g., media with alerting content and light, caffeine) may mask the cue for sleep onset and displace sleep in the evening in adolescents. A moderate shift in the DLMO can facilitate an earlier sleep onset, but changes in sleep behavior will not occur without modifications or limits (negotiated with the adolescent) on some of these external factors. Ongoing work in our laboratory targets both the circadian and psychosocial factors to advance sleep onset times as a means to advance sleep onset and increase sleep duration on school nights.

The short sleep for the 6 days (days 17–22) before coming into the laboratory may have attenuated the phase shifts we observed, similar to what has been observed in adults (Burgess and Eastman, 2005; Burgess, 2010) and adolescents (Crowley et al., 2018) who had their sleep duration experimentally restricted. The adolescents who need phase advancing bright light, however, will also likely have restricted sleep during the school week before a weekend with morning bright light. Therefore, our results are more likely to reflect the outcomes of those who need this type of phase advancing protocol the most as opposed to the minority of adolescents who are obtaining adequate sleep on school nights.

In summary, this study provides encouraging data to further develop a weekend phase advancing protocol for adolescents who have late bedtimes and short sleep on school nights. Our data also suggest that light stimuli used in previous reports with null findings may have been too short or timed too late to produce phase advance shifts of the adolescent circadian system. Supplementing our weekend morning light protocol with additional behavioral strategies to control light and dark exposure during the weekend and in the weeks that follow, as well as engaging adolescents in sleep behavior change are critical next steps.

## Data Availability Statement

The datasets generated for this study are available on request to the corresponding author.

## Ethics Statement

The studies involving human participants were reviewed and approved by the Institutional Review Board #1 at Rush University Medical Center. Written informed consent to participate in this study was provided by the participants’ legal guardian and the participant.

## Author Contributions

IM collected the data and drafted the manuscript. CE helped in designing the study and edited the manuscript. SC conceived of and designed the study, analyzed the data, and wrote the manuscript.

## Conflict of Interest

The authors declare that the research was conducted in the absence of any commercial or financial relationships that could be construed as a potential conflict of interest.

## Abbreviations

DLMO: dim light melatonin onset.
