# Supplementary material for: Circadian Phase Advances in Response to Weekend Morning Light in Adolescents With Short Sleep and Late Bedtimes on School Nights
Source: Front Neurosci. 2020 Feb 12;14:99. doi: 10.3389/fnins.2020.00099 (PMC7029701; doi:10.3389/fnins.2020.00099)
Supplement: Supplementary file 1 [file Image_1.PDF]

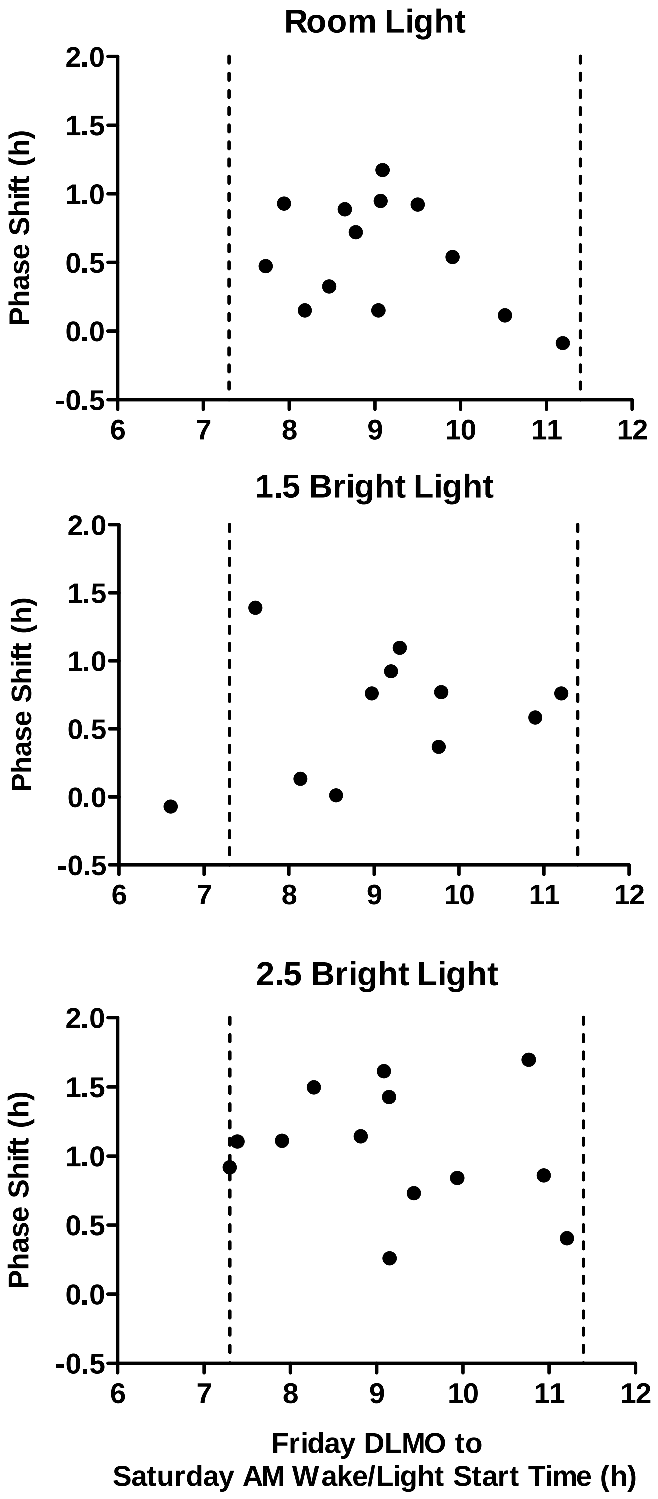

Figure S1. Weekend phase shifts (Friday DLMO (day 23) to Sunday DLMO (day 25)) plotted with respect to the time adolescents woke up and were exposed to the first room or bright light exposure on Saturday morning in the laboratory. Each point represents an individual participant. The adolescent PRC to bright light estimates that the largest phase advances occur when bright light exposure begins 7.3 to 11.4 h after initial DLMO (Crowley and Eastman, 2017). Vertical lines enclose the time when the largest phase advances are expected. All except one participant in the 1.5 h group woke and started light exposure within the phase advancing region.
