# Supplementary material for: Circadian Phase Advances in Response to Weekend Morning Light in Adolescents With Short Sleep and Late Bedtimes on School Nights
Source: Front Neurosci. 2020 Feb 12;14:99. doi: 10.3389/fnins.2020.00099 (PMC7029701; doi:10.3389/fnins.2020.00099)
Supplement: Supplementary file 2 [file Data_Sheet_1.PDF]

wavelength (nm)	spectral irradiance (μW/cm2)
380	0.226985169
385	0.2449198
390	0.312174665
395	0.440519365
400	0.935963537
405	24.32384281
410	4.068919327
415	4.634981107
420	6.893623654
425	9.808001134
430	13.2267901
435	103.6845834
440	20.00832231
445	22.19410542
450	24.94034574
455	27.23822029
460	28.91959191
465	30.15259777
470	30.60096354
475	30.71305498
480	33.51534102
485	57.16663518
490	57.72709239
495	45.22889666
500	34.13184394
505	23.93152277
510	20.45668808
515	17.93463064
520	15.63675609
525	13.67515587
530	12.38610429
535	20.7929624
540	95.83818251
545	195.0391083
550	72.85943699
555	19.50391083
560	7.229897978
565	5.828754959
570	5.419621198
575	18.38299641
580	37.77481579
585	42.9310221
590	40.68919327
595	29.48004912
600	23.65129416
605	19.50391083
610	154.6861893
615	84.62903835
620	37.43854147
625	37.94295296
630	42.59474778
635	17.8225392
640	15.30048177
645	14.74002456
650	16.64557907
655	12.72237861
660	12.27401285
665	12.10587568
670	8.79917816
675	8.014538069
680	7.454080862
685	7.566172303

690	8.294766673
695	6.277120725
700	3.850341016
705	10.42450406
710	17.14999055
715	6.277120725
720	2.275456263
725	1.972809371
730	1.731812772
735	1.445979596
740	1.496420744
